# Life‐history changes in the cold tolerance of the two‐spot spider mite *Tetranychus urticae*: applications in pest control and establishment risk assessment

**DOI:** 10.1111/phen.12262

**Published:** 2018-09-12

**Authors:** Nicola White, Jeffrey S. Bale, Scott A. L. Hayward

**Affiliations:** ^1^ Institute of Integrative Biology University of Liverpool Liverpool U.K.; ^2^ School of Bioscience University of Birmingham Birmingham U.K.

**Keywords:** Cold stress, diapause, environmental niche model, lethal temperature, lethal time, overwintering, pest control, supercooling point

## Abstract

Lethal time_50_ (LTime_50_) and lethal temp (LTemp_50_) are commonly used laboratory indices of arthropod cold tolerance, with the former often being employed to predict winter survival in the field. In the present study, we compare the cold tolerance of different life‐history stages (nondiapausing and diapausing females, as well as males and juveniles) of a major agricultural pest: the two‐spot spider mite *Tetranychus urticae* Koch (Acarina: Tetranychidae). Diapausing females from European populations of this species are shown to be freeze avoiding, supercooling to −23.6 ± 0.37  °C and with an LTemp_50_ of −23.2 °C. However, nondiapausing females [supercooling point (SCP) –19.1 ± 0.49 °C, LTemp_50_ –14.32 °C], males (SCP –21.27 ± 0.52  °C, LTemp_50_ –16 °C) and juveniles (SCP –25.34 ± 0.29 °C, LTemp_50_ –18.3 °C) are subclassified as strongly chill tolerant juveniles. LTime_50_ is 148.3 days for non‐acclimated diapausing females, whereas nondiapausing females, males and juveniles reach 50% mortality by 21.7 days. When individuals are acclimated at 10 °C for a period of 7 days, no effect is found. Cold tolerance is suggested to be a major contributor to the successful spread of T. urticae across temperate countries, although it is dependent on a diapause trait, suggesting a potential target for control. Winter field trial data from diapausing females indicate that LTime_50_ is a reliable indicator of winter survival even within diapause, supporting the use of these indices as a valuable component within environmental niche models for the prediction of future pest invasions.

## Introduction

Increasing evidence suggests that understanding the thermal of a species tolerance can enhance predictions of their potential to invade new geographical regions (Terblanche *et al*., 2006, 2007; Mitchell & Hoffmann, 2010; Alford *et al*., 2012). Ecological and environmental factors contribute to the environmental niche model (ENM), which takes the known distribution of a potentially invasive species and correlates it with these variables to predict population establishment in new regions (Elith & Leathwick, 2009; Jiménez‐Valverde *et al*., 2011; Hill *et al*., 2013). Accordingly, laboratory and field based stress physiology experiments often form the basis of establishment risk assessments with respect to providing a license for the use of novel biological control agents (BCAs) in non‐native countries (van Lenteren *et al*., 2006). This is equally relevant for pest species. Studies on the dark sword grass moth (*Agrotis ipsilon*) suggest that a lack of cold hardiness may explain why this pest is unable to establish a permanent population in the U.K. (Bale, 2002), whereas increased high temperature tolerance in the mite *Halotydeus destructor* corresponds with predicted niche shifts and extended distributions of this species in Australia (Hill *et al*., 2013).

The two‐spot spider mite *Tetranychus urticae* Koch (Acarina: Tetranychidae) is a well‐known, highly polyphagous pest with significant economic impact, causing direct damage through feeding and reducing photosynthetic activity (Rabbinge, 1985). In the U.K., *T. urticae* is particularly dominant in intensive, high‐yield cropping systems such as tomatoes, strawberries and chrysanthemums (Sances *et al*., 1982; Easterbrook *et al*., 2001; Gorman *et al*., 2002). Although, similar to many pests, this species favours glasshouse crops, it is also a major pest of open crop systems (e.g. soybean and cotton) (Ay & Gürkan, 2005; Razmjou *et al*., 2009). Originally described in Europe, *T. urticae* has gained a worldwide distribution throughout temperate regions, as well as the subtropics (CABI, 2015). It is a good example of a species that posed no major economic threat until the extensive use of pesticides eradicated their natural predators, whereas *T. urticae* developed resistance (Gerson & Weintraub, 2007). This rapid development of pesticide resistance is the dominant factor contributing to the success of this mite; however, the ability to survive temperate winters is also likely to have played an important role in their range expansion.

There are multiple laboratory‐based experiments assessing a species' cold tolerance strategy and ability to survive winter (van Lenteren *et al*., 2006). Supercooling point is the temperature at which an individual is no longer able to avoid freezing. At the most basic level, where the lower lethal temperature (LTemp) is above the supercooling (freezing) point (SCP), the species is considered to be freeze avoiding (FA), whereas, if the LTemp is below the SCP, then that species is classified as freeze tolerant (FT) (Bale, 1993). However, this simple two‐way classification masks a much more complex situation, where mortality is experienced at temperatures often well above the SCP, depending on the species, developmental stage, sex and physiological history, etc. (Bale, 1996). There is a wealth of evidence indicating that different developmental stages of the same species can differ markedly in both their cold tolerance (Block *et al*., 1990; Lindsay *et al*., 1998; Paur & Gray, 2011), even whether they are FA or FT (Ansart & Vernon, 2004; Bouchard *et al*., 2006). Equally, males and females can demonstrate contrasting abilities to survive cold stress (Helden & Dixon, 2002; Yonow *et al*., 2004; Knapp & Saska, 2012), although this is often neglected in most studies (Renault *et al*., 2002).

Cold acclimation (the exposure of individuals to sublethal temperatures) and diapause are also shown to influence low temperature tolerance in many arthropod species (Denlinger, 1991; Morewood, 1993; Kandori *et al*., 2006; Takano, 2014) and there is good evidence for similar molecular processes underpinning both these processes (Ding *et al*., 2003; Hahn & Denlinger, 2011; Khodayari *et al*., 2013; Teets & Denlinger, 2013). Adult female diapause is assumed to be the only overwintering stage of *T. urticae* from a wild population in Iran, as shown by their significantly increased cold hardiness in the diapause state. This is indicated by an SCP of −25.3 °C compared with −19.6 °C for nondiapausing females and an LTemp_50_ of –19.7 °C versus −13.3 °C, respectively (Khodayari *et al*., 2012). Consequently, the disruption of diapause, which is induced by a shortening of the photoperiod, is considered as a potential pest control strategy (Krysan, 1990; Doucet *et al*., 2007). However, interfering with the diapause trait becomes less relevant if other developmental stages have the capacity to survive winter conditions. Assessing survival outside of diapause is also pertinent in light of climate change because there is increasing evidence that warming temperatures may disrupt the diapause cycle, leaving nondiapause stages vulnerable to winter cold, which in turn can influence species distribution (Bale & Hayward, 2010; Coleman *et al*., 2014). Thus, gaps remain in our understanding of *T. urticae* cold tolerance, given that previous studies do not investigate the males, females or juveniles, nor do they investigate populations from more northerly locations likely to encounter colder winter conditions. Lower thermal limits can certainly differ significantly across latitudinal populations of the same species, both within and outside of diapause (Saunders & Hayward, 1998; Addo‐Bediako *et al*., 2000; Sunday *et al*., 2011).

Winter field trials are also yet to be conducted with *T. urticae* and provide a key indicator of long‐term survival under low temperature conditions as well as potential establishment risk. However, although field trials can provide the most realistic assessment of winter survival, they can be difficult to conduct successfully (Hatherly *et al*., 2005). Problems include the requirement for large numbers of individuals, which, for predatory arthropods, must be kept separate to avoid intraguild predation. Such trails are also typically very time consuming, labour intensive and can encounter issues with highly variable conditions between years. Collectively, these factors make it unfavourable for commercial companies to undertake extensive field trials and so alternative laboratory indices of cold tolerance that are good predictors of winter survival have been sought. Lethal time (LTime) is considered the laboratory equivalent of a winter field trial, where individuals are exposed to low temperatures (most commonly 5, 0 or −5 °C) to assess the length of time needed to experience mortality, often reported as 50% mortality (LTime_50_) (McDonald *et al*., 1997; Jing & Kang, 2003; Maes *et al*., 2015).

Using a linear regression analysis, Hatherly *et al*. (2005) propose LTime_50_ at 5 °C as the optimal laboratory‐based experiment for European Union (EU) commercial companies to undertake when assessing the capacity for winter survival in temperate countries because a strong correlation between LTime_50_ and maximal field survival is identified across a range of species. This results in an accumulated dataset of LTime_50_ at 5 °C for a range of species (latest version: Coombs & Bale, 2014; dataset available on request) and is supported as the basis of U.K. legislation with respect to assessing establishment risk of non‐native glasshouse BCAs (Bale, 2011). This LTime_50_ at 5 °C regression analysis is already reported to be used in the assessment of field survival probability for several species, including the invasive *Harmonia axyridis*, as well as several phytoseiid BCAs: *Amblyseius swirskii*, *Typhlodromips montdorensis*, *Phytoseiulus longipes* and *Neoseiulus californicus* (Hatherly *et al*., 2005; Allen, 2010; Raak‐van den Berg *et al*., 2012). However, traits such as diapause and acclimatory responses that can increase the thermal tolerance of some species (Colinet & Hoffmann, 2012; Khodayari *et al*., 2012; Foray *et al*., 2013; Denlinger & Armbruster, 2014) are not always considered.

Against this background, the present study aimed to investigate the capacity of diapause and nondiapause stages (including males) of European *T. urticae* populations to survive different U.K. winter conditions, at the same time as comparing the efficacy of different laboratory‐based cold tolerance indices (LTemp and LTime) with respect to predicting winter survival. For the first time, the present study also aims to include a diapausing species within the Hatherly *et al*. (2005) regression and discusses the capacity of *T. urticae* to maintain a cosmopolitan distribution. We discuss the suitability of LTime_50_ at 5 °C regression as a key component of the ENM, and subsequently as a component of EU BCA applications.

## Materials and methods

### *Rearing*

The population of *T. urticae* was provided by Biobest NV (Belgium) from a laboratory population originally sourced from a range of European sites by Biobest and the University of Warwick. Although this laboratory population has been in culture for many years (at least 8 years), it has regularly been supplemented with wild caught samples and thus is representative of the species' cosmopolitan distribution and retains the diapause trait expressed in all wild populations. Nondiapausing samples (juveniles, males and females) were reared under quarantine conditions under an LD 18 : 6 h photocycle at 23 °C (= non‐acclimated condition) on dwarf French bean plant *Phaseolus vulgaris* (Fabales: Fabaceae). Diapause females were reared using the methods described by Singh & Clark (1993). Gravid females were placed on uninfested dwarf French bean plants and allowed to oviposit for 24 h under an LD 24 : 0 h photocycle at 23 °C to maximize oviposition. The females were removed and the plant was transferred to an LD 6 : 18 h photocycle at 20 °C because both photoperiod and temperature influence diapause induction. Samples were then maintained under an LD 6 : 18 h photocycle at 20 °C throughout diapause. After 3–4 weeks, diapausing females were identified by their orange colour. Acclimation is the exposure of an individual to a sublethal temperature that aims to induce a physiological change aiding survival. We acclimated individuals at 10 °C for a period of 7 days with fresh cut dwarf French bean leaves. Non‐acclimated controls were transferred to stress treatment direct from 23/20 °C, unless otherwise stated. The juvenile developmental stage included both protonymphs and deutonymphs, as a result of difficulty in distinguishing between these stages, and were distinguished from adults by only having six legs.

### *SCP*

SCPs were determined *sensu* Bale *et al*. (1984). Individual mites were adhered to type K exposed wire thermocouples with a minimal amount of OecoTak (Oecos, U.K.). The thermocouple was placed inside a size 3 Beem capsule (Agar Scientific Ltd, U.K.), inside a test tube suspended in a programmable alcohol bath (Haake Phoenix II; Artisan Technology Group, Champaign, Illinois). The thermocouples were connected to a computer running picolog recorder (Pico Technology). The temperature was ramped at a rate of 0.5 °C min^−1^ from 23 °C (or the acclimated temperature, 10 °C) to −30 °C. The SCP was detected by the exothermic release. Mean SCPs for acclimated and non‐acclimated treatments were calculated for 30 mites (three replicates of 10 mites).

### *LTemp*

Individual mites were separated into size 3 Beem capsules and split into three groups of 10. Each group of 10 capsules were placed in a test tube, which was suspended in a programmable alcohol bath. Samples were ramped (0.5 °C min^−1^) from the rearing temperature (23/20 °C) or acclimation temperature (10 °C) to a range of predetermined temperatures expected to cause 0–100% mortality (ranging from −6 to −28 °C. Mites were held for 10 min at the stressful temperature before being ramped back (0.5 °C min^−1^) to the rearing (23/20 °C) or acclimation (10 °C) temperature. On return to the rearing temperature, the mites were placed in groups of 10 in a sealed, ventilated container with a non‐infested *P. vulgaris* leaf and moisture source. For the control, individuals were held within identical containers suspended in an alcohol bath programmed at their rearing temperature and left for 3 h before being returned to standard rearing conditions. Mortality after 72 h for all life‐history stages (adult males, nondiapausing females, diapausing females and juveniles) was assessed for each temperature, using three replicates of *n* = 10.

### *LTime and field trial*

Mites were placed into arenas with a dwarf French bean leaf and moisture source before being transferred to 10 °C for 1 h, prior to cold exposure, to avoid cold shock. This was not required for acclimated mites. To measure LTime_50_, samples were exposed to 5 °C for a series of time periods (between 1 and 50 days, except for diapausing females, where it was extended to > 200 days), predetermined to cause 0–100% mortality.

For the winter field trial, all samples were placed in sealed ventilated plastic boxes with access to food at a secure and sheltered location in a field in Birmingham, U.K. (52.4°N). Microclimate conditions within these containers were recorded continuously using TinyTag temperature dataloggers (Gemini Data Loggers, U.K.). Dwarf French bean leaves were replaced periodically. At set time intervals during winter, samples were removed to assess mortality, returning them to rearing conditions (23 °C) after 1 h at 10 °C (to avoid heat shock) with mortality assessed after 72 h. The control exposure was set up as described but maintained at 23 °C for 7 days. Each experiment was conducted with three replicates of *n* = 10.

### *Statistical analysis*

All statistical tests were carried out in Minitab, version 17 (Minitab Inc., State College, Pennsylvania). All data were tested for normality using Kolmogorov–Smirnov. Levene's method and the multiple comparisons method were used to assess equal variances of errors, where necessary.

LTemp and LTime data were analyzed using Probit analysis (Finney, 1971), reporting values that resulted in 50% and 90% mortality. Significance was identified by non‐overlapping fiducial limits. SCP data required a nonparametric Kruskal–Wallis test with a post‐hoc Mann‐Whitney *U*‐test. Winter field trial data were analyzed using a binary logistic regression (Harrell, 2015) to detect any significant differences between the cohorts and treatments. Data were assessed for goodness‐of‐fit using Pearson's chi‐squared statistic. *P* < 0.05 was considered statistically significant.

## Results

### *SCP*

All individuals were found to be freeze intolerant, although there were significant differences between SCPs across acclimated and non‐acclimated life stages (*H* = 119.56, d.f. = 7, *P <* 0.01) (Table 1). Acclimation significantly lowered the SCP of all adult samples but not juveniles (*W* = 951.5, *P =* 0.59). Males had lower mean SCPs (−21.3 ± 0.5 °C) than nondiapause females (−19.1 ± 0.5 °C ).

**Table 1:** Mean ± SE and range of supercooling points of non‐acclimated and acclimated T. urticae, juveniles, males and nondiapausing or diapausing females.

Experimental group	*N*	Mean ± SE (°C)	Range (°C)
Non‐diapausing females			
Non‐acclimated	30	−19.1 ± 0.5^*a*^	−12.1 to −23.2
Acclimated	30	−22.0 ± 0.6^*b*^	−12.0 to −26.1
Males			
Non‐acclimated	30	−21.3 ± 0.5^*b*^	−11.8 to −26.3
Acclimated	30	−24.0 ± 0.3^*c*^	−18.9 to −26.9
Juveniles			
Non‐acclimated	30	−25.3 ± 0.3^*df*^	−22.5 to −28.6
Acclimated	30	−25.3 ± 0.4^*de*^	−19.0 to −28.9
Diapausing females			
Non‐acclimated	30	−23.6 ± 0.3^*c*^	−18.5 to −2.8.3
Acclimated	30	−25.4 ± 0.4^*ef*^	−22.0 to −28.7

Means that do not share a lowercase letter are significantly different (*n* = 30).

### *LTemp*

**Figure 1:**
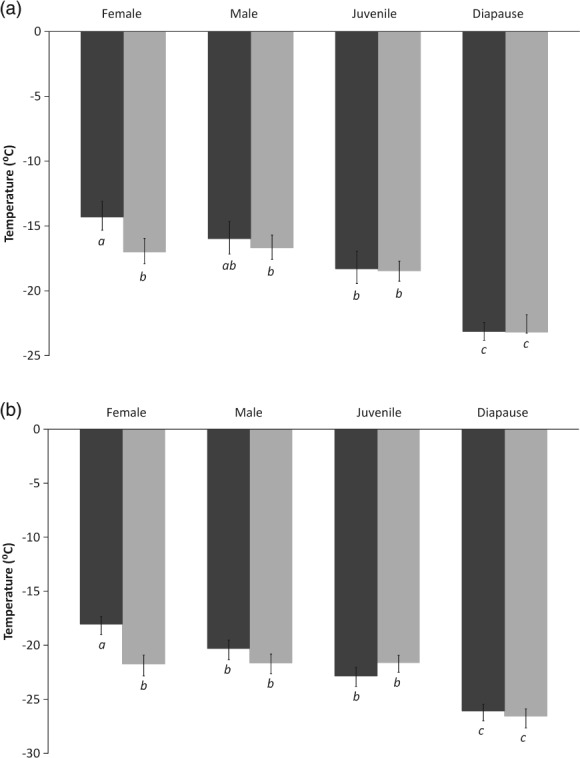
Lethal temperature (± 95% fiducial limits) resulting in 50% mortality (a) and 90% mortality (b) of non‐acclimated (dark grey) and acclimated (light grey) T. urticae females, males, juveniles and diapausing females. Means with the same lowercase letter are not significantly different (*p* > 0.05).

LTemp resulting in 50% (LTemp_50_) (Fig. 1a) and 90% (LTemp_90_) (Fig. 1b) mortality of the acclimated and non‐acclimated life stages indicated that non‐acclimated nondiapausing adult females were the least cold tolerant (LTemp_50_ = −14.2 °C), with acclimation significantly enhancing their cold survival (LTemp_50_ = −17.2 °C; non‐overlapping fiducial limits) (Fig. 1). Acclimation did not have a significant effect on the LTemp of any other treatment groups and the cold tolerance of males and nondiapausing females did not differ significantly (overlapping fiducial limits) (Fig. 1). Diapausing females (non‐acclimated and acclimated) had a significantly lower LTemp_50_ than all other groups (−23.2 °C for both; non‐overlapping fiducial limits) (Fig. 1). The LTemp_90_ values of all groups were within 3.6 °C of their corresponding SCP. No mortality was recorded in controls.

### *LTime*

**Figure 2:**
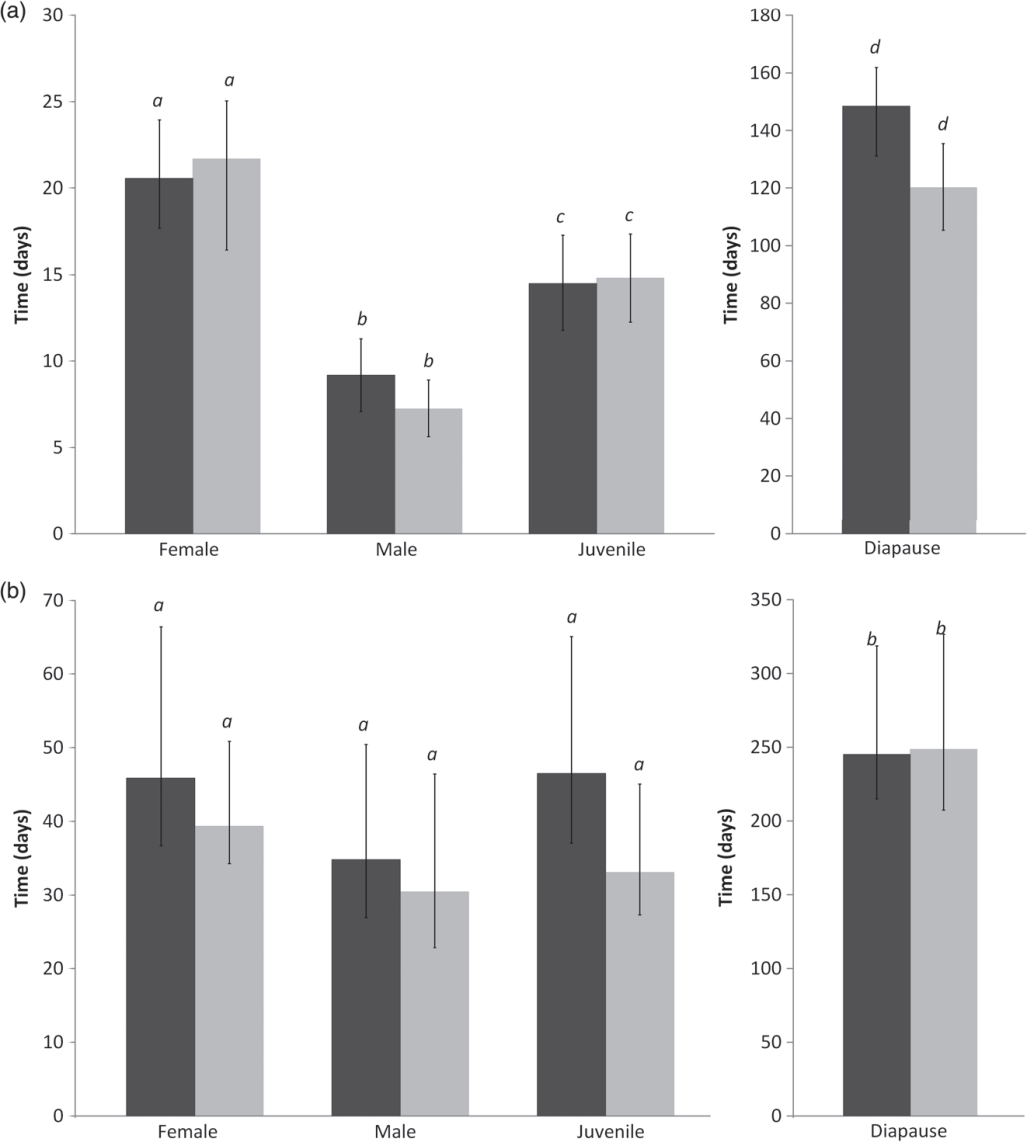
Lethal time for 50% mortality (a) and lethal time for 90% mortality (b) (± 95% fiducial limits) at 5 °C for various non‐acclimated (dark grey) and acclimated (7 days at 10 °C) (light grey) T. urticae. Means with the same lowercase letter are not significantly different (*p* > 0.05).

The LTime_50_ data indicated significant differences in cold tolerance between developmental stages (non‐overlapping fiducial limits) (Fig. 2). Mortality at 5 °C increased most rapidly in males, with 50% mortality before 10 days, followed by juveniles (mean LTime_50_ of approximately 15 days) and nondiapausing females (mean LTime_50_ of approximately 20 days). Both non‐acclimated and acclimated diapausing females had mean LTime_50_ values beyond 120 days. Acclimation for 7 days at 10 °C did not significantly increase the LTime_50_ of any treatment group (overlapping fiducial limits) (Fig. 2). The longest LTime_90_ values were also for diapausing females and these were significantly longer than juveniles, males or nondiapausing females (non‐overlapping fiducial limits) (Fig. 2b). LTime_90_ values for these other developmental stages did not differ significantly from each other. No mortality was recorded in the control.

### *Field trial*

**Figure 3:**
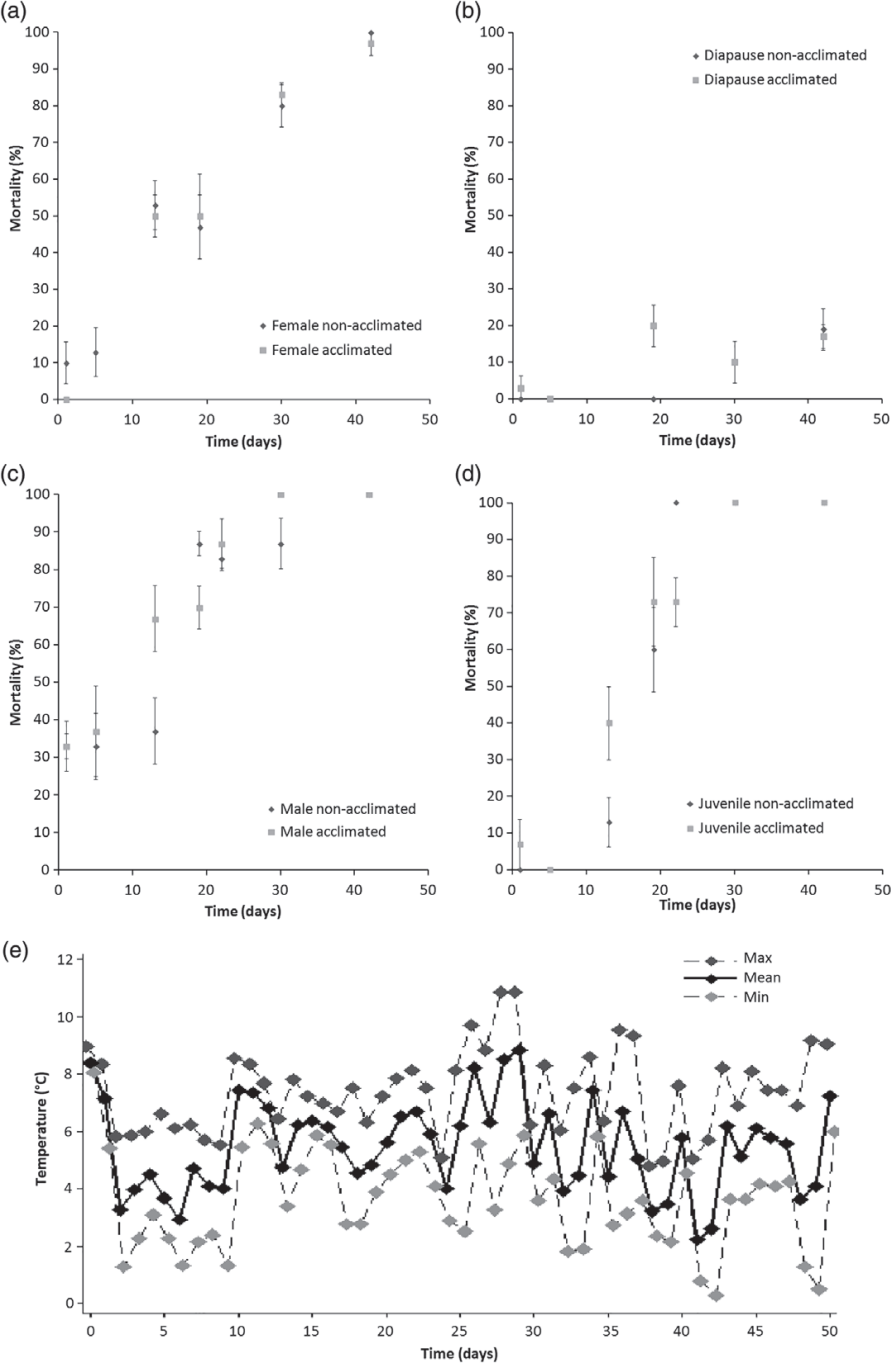
Mortality (± SE) during winter of (a) female, (b) diapausing females, (c) male and (d) juvenile; non‐acclimated (dark grey diamonds) and acclimated (light grey squares) T. urticae in a field in Birmingham, U.K., from 17 November 2014. The minimum, maximum and mean temperature exposure during this period are shown in (e).

The mortality of *T. urticae*, non‐acclimated and acclimated individuals was recorded in the field from 17 November 2013 until 29 December 2013 (Fig. 3a–d), in addition to daily minimum, maximum and average field temperatures (Fig. 3e). The mean temperature for the field trial period was 5.5 ± 0.1 °C and the minimum temperature was 0.1 °C. Mortality of all treatment groups increased with duration of field exposure (χ^*2*^ = 471.67, *P* < 0.01). There were significant differences in survival between non‐acclimated nondiapausing females, males, juveniles and diapausing females (χ^*2*^ = 426.11, *P* < 0.01). Mortality reached 100% for non‐acclimated males, juveniles and nondiapause females within 42 days. Non‐acclimated diapausing females experienced only 19% mortality in the same time period. Unfortunately, as a result of mould growth in the diapause arenas, the field trial was ceased at day 60 and only data until day 42 were included in the analysis. Acclimation did not enhanced the winter survival of any developmental group (χ^*2*^ = 4.06, *P* > 0.05). No mortality was recorded in the control.

## Discussion

As a foreign, invasive species that has become a major agricultural pest, the ability of *T. urticae* to develop resistance to pesticides, their response to plant toxins, changes in plant host ranges and genetic differentiation between populations are all well studied (Navajas *et al*., 2000; Boom *et al*., 2003; Li & Romeis, 2010; Van Leeuwen *et al*., 2010; Dermauw *et al*., 2013). By contrast, the thermal biology of this species is less well characterized, although equally important, because, in temperate regions, winter temperatures can be considered as one of the most important factors affecting the establishment potential of arthropods (DeBach, 1964; van Lenteren *et al*., 2006). Thus, investigations of cold tolerance can be used to assess establishment risk and potential for range expansion, as well as the survival of winter populations to help predict pest problems in the subsequent year (Samways *et al*., 1999; van Lenteren *et al*., 2006; Bale, 2010; Berkvens *et al*., 2010). The effects of temperature on nondiapausing and diapausing female *T. urticae* from Iran are reported by Khodayari *et al*. (2012), followed by investigations into the underlying biochemical response mechanisms (Khodayari *et al*., 2013). Acclimation at 5 °C for 10 days is seen to enhance the acute cold tolerance (LTemp_50_) of diapausing females in particular, and nondiapause females to a lesser degree, whereas 0 °C acclimation has a limited effect. The present study extends this to include an assessment of cold tolerance in a laboratory population of mixed European origins of *T. urticae* and responses to acclimation at 10 °C, as well as a characterization of the cold tolerance of both juveniles and males. In addition, we investigate more ecologically relevant long‐term cold exposures in the laboratory (LTime), aiming to assess the efficacy of this cold tolerance index to predict winter survival via direct comparison with winter field trials.

The results of the present study concur with those of Khodayari *et al*. (2012) in that all developmental stages of *T. urticae* are chill tolerant, with SCPs ranging from −19 °C for non‐acclimated nondiapause females to −25.4 °C for acclimated diapausing females, and with LTemp_50_ spanning −14.3 °C (−13.3 °C in Khodayari *et al*., 2012) for non‐acclimated nondiapause females to −23.1 °C for acclimated diapause females. Interestingly, acclimation at 10 °C significantly lowers the SCPs of all adults (Table 1), although it only lowers the LTemp of nondiapause females. This compares with the results of the study by Khodayari *et al*. (2012) where 10‐day acclimation at 5 °C has no effect on diapause SCP but improves all other SCP and LTemp_50_ conditions. This reinforces the idea that SCP temperatures are not always a useful indicator of cold tolerance (Bale, 2002), although it does demonstrate that 5 and 10 °C are sufficient to induce some kind of physiological cold response. However, this response was insufficient to affect survival during long‐term cold exposures (Figs 2 and 3).

### *LTime_50_ as an indicator of thermal tolerance*

The LTime at 5 °C data indicate that males are the least cold hardy, followed by juveniles, then nondiapause females, with no significant improvement as a result of acclimation (Fig. 2). Combining the LTime, LTemp and SCP data, it is predicted that males, nondiapause females and juveniles would not survive a U.K. winter, which is confirmed in a winter field trial (Fig. 3).

**Figure 4:**
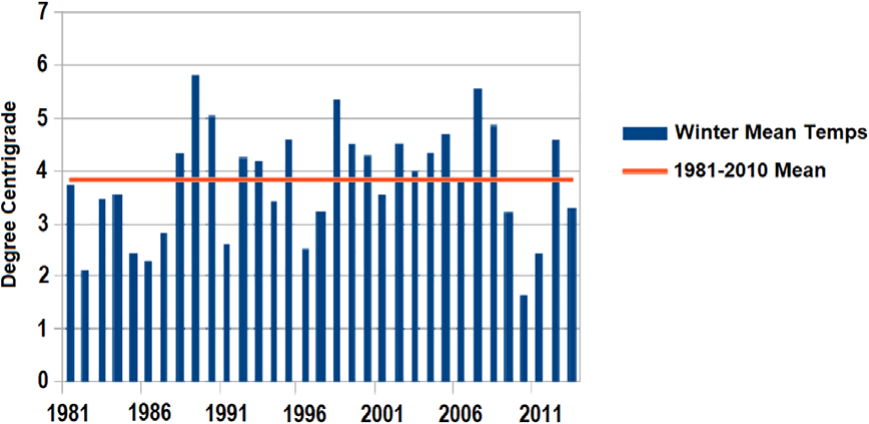
Average winter temperatures in the U.K. from December 1980 to February 2013. Not shown are the temperatures for winter 2013/2014, with an average of 5.2 °C, and winter 2014/2015, with an average of 3.9 °C (Met Office). [Colour figure can be viewed at http://wileyonlinelibrary.com].

Diapause in several Phytoseiidae species is confirmed to increase the cold tolerance of females (Denlinger, 1991; Morewood, 1993). The present study also finds that diapausing females of *T. urticae* are the most cold tolerant life cycle stage with an LTemp_50_ of −23.2 °C, an SCP of −23.6 °C and an LTime_90_ at 5 °C of 245.1 days. Also, during a winter exposure, only 19% mortality is recorded in the time required for 100% mortality to occur in juveniles, males and nondiapausing females. The winter trial is carried out during a mild U.K. winter (average of 5.5 °C) (Fig. 4) compared with the U.K. average of 3.8 °C (averaged data from 1981–2010, Met Office) and, as such, it can be assumed that, without a diapause trait, *T. urticae* would not survive most U.K. winter periods. This further reinforces the fact that SCPs are rarely a good indicator of cold tolerance (Salt, 1961; Sømme, 1982; Knight *et al*., 1986; Bale, 1996; Renault *et al*., 2002) because juvenile *T. urticae* have lower SCPs than diapausing females.

Field trials are often proposed as the most reliable method for determining cold tolerance and overwintering abilities. There are limitations, however, where individuals must be in conditions that allow monitoring and assessment of survival, which can reduce the ecological relevance. For example, individuals may not be able to select their most optimal overwintering site such as ground cover or under bark (Pfiffner & Luka, 2000; Overgaard *et al*., 2011). These disadvantages can be minimized by including food and water and, where needed or able, mimicking natural shelters. The primary advantage, however, is that the species are directly experiencing winter conditions, with natural temperature fluctuations and light cycles that are difficult to accurately replicate within a laboratory.

**Figure 5:**
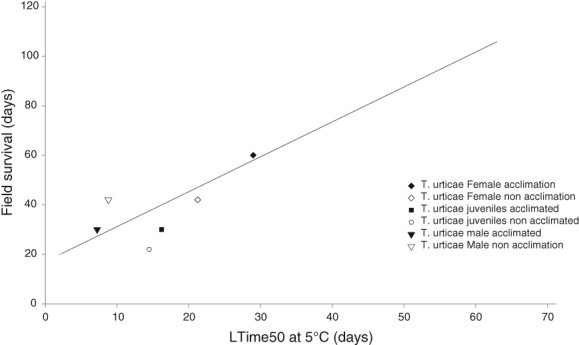
Relationship between maximum field survival (days) and lethal time for 50% mortality (LTime_50_) at 5 °C (days) for the different acclimated and non‐acclimated life stages of Tetranychus urticae. Modified from Coombs & Bale, 2014. Only showing data for T. urticae.

As a surrogate measure, LTime_50_ at 5 °C data can provide applicable indices of cold tolerance that are less labour intensive and time consuming to collect. These often show a good correlation with winter field survival (Hatherly *et al*., 2005), as found to be the case for juvenile, male and nondiapausing female *T. urticae* (Fig. 5). Unfortunately, because the winter field trial is terminated before diapausing females reach 100% mortality, they cannot be plotted onto this regression. However, using the regression equation (Fig. 5) combined with an LTime_50_ of 143.8 days, we predict that diapausing females could survive in the field for around 220 days (i.e. much longer than a U.K. winter). Diapausing females have an LTime_90_ of 245.1 days (Fig. 2b), which complements the predicted survival time from the regression analysis. It can therefore be implied that an LTime_50_ at 5 °C could also be applied with respect to predicting the field survival of diapausing stages in other species.

Although acclimation at 10 °C does not enhance the survival of *T. urticae* in either laboratory experiment, it is important to still consider acclimatory responses when incorporating other species in Fig. 5, especially because there is some evidence that 5 °C may enhance the long‐term survival of *T. urticae* (Khodayari *et al*., 2012). Acclimatory responses are also identified in many other important pests, such as the sweet potato weevil *Cylas formicarius* and the melon thrip *Thrips palmi* (McDonald *et al*., 2000; Kandori *et al*., 2006), as well as the generalist predator *Euseius gallicus* (N. White, S. A. L. Hayward & J. S. Bale, unpublished data). With an LTime_50_ of 40.5 days for acclimated *E. gallicus* adults, the maximal field survival is predicted to be 80 days; however, the survival is reported to only reach 50% mortality after 116 days in the field. Thus, for acclimated *E. gallicus*, the graph does not accurately predict winter survival, highlighting a potential limitation of this regression in some cases.

### *Potential underlying molecular mechanisms of cold tolerance*

Metabolic differences between nondiapausing and diapausing females are likely to account for the significant differences in winter survival, particularly the biochemical events related to the cold hardening process characterized in other arthropod species (Li *et al*., 2001; Koštál *et al*., 2003; Michaud & Denlinger, 2007; Hahn & Denlinger, 2011). Several studies report considerable overlap in the molecular processes underpinning diapause and cold acclimation (Ding *et al*., 2003; Hahn & Denlinger, 2011; Teets & Denlinger, 2013). Understanding the metabolic differences could provide an essential starting point for interrupting the diapause trait as a form of biological control in temperate countries in *T. urticae* (Krysan, 1990; Doucet *et al*., 2007).

### *ENM*

All species possess a maximum level of thermal tolerance, which in combination with other factors, limits their distributions and opportunities for future range expansion (Bale, 1996). Using the known distribution of an invasive species and correlating this with environmental variables is proposed as a pre‐emptive approach for pest control (Hill *et al*., 2013). For example, the Asian Longhorn beetle (*Anoplophora glabripennis*) began its invasive spread of North America in the 1990s. A predictive model (ecological niche model) by Peterson & Vieglais (2001) shows that the species had the potential to establish in eastern North America but not along the Pacific coast. So far, there have been no reports of this species along the Pacific coast (EPPO, 2014), supporting the model's predictions. The same model predicted *Anoplophora malasiaca* as a potential invasive pest, which has now been added to the EPPO watch list, with a non‐native population established in Italy (Colombo & Limonta, 2001; EPPO, 2001).

The ENM primarily uses the environmental variables a species experiences (e.g. precipitation and land cover) to predict the potential to invade new regions (Elith & Leathwick, 2009; Jiménez‐Valverde *et al*., 2011; Hill *et al*., 2013). Although temperature is only one of the environmental factors contributing towards the ENM, it is an important one that can be studied with relative ease. *Tetranychus urticae* is here classified as a freeze avoiding species (chill tolerant for nondiapausing stages) (Bale, 1993) with LTime_50_ at 5 °C accurately predicting winter field survival, as is shown in many other species (Coombs & Bale, 2014). The thermal tolerance of *T. urticae* correlates with its cosmopolitan invasion of temperate countries but not the more extreme latitudes (e.g. there are populations in Canada but not Alaska) (CABI, 2015). Other important factors in the ENM include dispersal ability, competition, predation and access to hibernation sites (Samways *et al*., 1999; Baker *et al*., 2000), although these are rarely studied in detail and can be labour‐intensive to undertake. Temperature is clearly a primary environmental parameter determining arthropod survival and distribution. As such, LTime_50_ at 5 °C, which is typically not labour‐intensive, is supported as an essential component for consideration in all ENM assessments. By proposing the use of one reliable experiment, the procedures may be refined and used consistently across all research groups, reducing any potential inaccuracy when comparing different species.

It is worth noting that, although 5 °C is a common temperature within a U.K. winter, for other temperate countries experiencing lower average temperatures, 0 or −5 °C may be a better suited temperature for LTime assessments. Hence, although LTime_50_ may be a valuable index in developing ENMs, the temperature at which it is assessed needs to be relevant to the full range of winter conditions experienced by a given species. Certainly, for the U.K., it appears that winter field trials could be replaced by simply assessing LTime_50_ at 5 °C when investigating the invasive potential of a species, either pest or BCA.

### *Conclusions*

*Tetranychus urticae* is a freeze avoidant (diapause)/chill tolerant (nondiapause stages) species. The ability of this species to diapause is likely a major contributing factor to the successful widespread inhabitation of temperate countries, combined with their rapid life cycle, inbreeding habits and arrhenotokous reproduction and pesticide resistance. Without the diapause trait, *T. urticae* cannot survive a temperate winter, suggesting disruption of the diapause trait as a potential control strategy for this species. LTime_50_ at 5 °C is supported as the optimal laboratory‐based representation of field relevant cold stress and is proposed as an essential component for accurate ENMs, with 0 or −5 °C being suggested for countries experiencing lower average temperatures than the U.K.
